# Efficacy of Pulsatile Flow Perfusion in Adult Cardiac Surgery: Hemodynamic Energy and Vascular Reactivity

**DOI:** 10.3390/jcm10245934

**Published:** 2021-12-17

**Authors:** Mikhail Dodonov, Francesco Onorati, Giovanni Battista Luciani, Alessandra Francica, Maddalena Tessari, Tiziano Menon, Leonardo Gottin, Aldo Domenico Milano, Giuseppe Faggian

**Affiliations:** 1 Cardiac Surgery, Department of Surgery, University of Verona Medical School, 37126 Verona, Italy; mikhail.dodonov@aovr.veneto.it (M.D.); francesco.onorati@univr.it (F.O.); giovanni.luciani@univr.it (G.B.L.); maddalena.tessari@univr.it (M.T.); tiziano.menon@aovr.veneto.it (T.M.); giuseppe.faggian@univr.it (G.F.); 2 Anesthesiology, Department of Surgery, University of Verona Medical School, 37126 Verona, Italy; leonardo.gottin@univr.it; 3 Cardiac Surgery, Department of Emergency and Organ Transplants, University of Bari Medical School, 70124 Bari, Italy; aldo.milano@uniba.it

**Keywords:** pulsatile perfusion, cardiopulmonary bypass, hemodynamic energy, endothelial integrity

## Abstract

**Background:** The role of pulsatile (PP) versus non-pulsatile (NP) flow during a cardiopulmonary bypass (CPB) is still debated. This study’s aim was to analyze hemodynamic effects, endothelial reactivity and erythrocytes response during a CPB with PP or NP. **Methods:** Fifty-two patients undergoing an aortic valve replacement were prospectively randomized for surgery with either PP or NP flow. Pulsatility was evaluated in terms of energy equivalent pressure (EEP) and surplus hemodynamic energy (SHE). Systemic (SVRi) and pulmonary (PVRi) vascular resistances, endothelial markers levels and erythrocyte nitric-oxide synthase (eNOS) activity were collected at different perioperative time-points. **Results:** In the PP group, the resultant EEP was 7.3% higher than the mean arterial pressure (MAP), which corresponded to 5150 ± 2291 ergs/cm^3^ of SHE. In the NP group, the EEP and MAP were equal; no SHE was produced. The PP group showed lower SVRi during clamp-time (*p* = 0.06) and lower PVRi after protamine administration and during first postoperative hours (*p* = 0.02). Lower SVRi required a higher dosage of norepinephrine in the PP group (*p* = 0.02). Erythrocyte eNOS activity results were higher in the PP patients (*p* = 0.04). Renal function was better preserved in the PP group (*p* = 0.001), whereas other perioperative variables were comparable between the groups. **Conclusions:** A PP flow during a CPB results in significantly lower SVRi, PVRi and increased eNOS production. The clinical impact of increased perioperative vasopressor requirements in the PP group deserves further evaluation.

## 1. Introduction

Despite continuous technological improvements in cardiac anesthesia, perfusion technique and myocardial protection, the conventional cardiopulmonary bypass (CPB) still remains a non-physiological scenario [1]. The role of pulsatile flow during short-term and long-term extracorporeal support is still controversial. Until now, the precise mechanisms underlying the physiological effects of pulsatile and non-pulsatile perfusion are not well understood. The shortcoming of a non-pulsatile flow consists of lower mechanical energy transmission to the vascular wall that results in decreased endothelial shear stress. The mechanical unloading of arterial baroreceptors leads to a marked increase in sympathetic activity with further progressive vasoconstriction and worsening of peripheral blood flow [2]. At the same time, the lower mechanical energy of non-pulsatile flow reduces the synthesis of shear-responsive, endothelial-derived vasodilators such as nitric oxide. It also contributes to the progressive capillary collapse, microcirculatory shunting and finally leads to tissue hypo-perfusion [2].

Pulsatile flow seems more physiologic as it mimics the natural blood flow produced by the human heart. The theoretical benefits of pulsatile perfusion consist of additional energy transmission to the vascular endothelium [3,4]. It results in higher endothelial shear stress, the augmented release of vasodilative molecules [5,6] and lower systemic vascular resistance with, consequently, better organs perfusion during and just after a pulsatile CPB [5,7,8,9].

The perioperative detection of endothelial integrity markers and vascular reactivity monitoring could reveal the influence of either flow type on the quality of the internal organs’ perfusion and help to predict clinical outcomes. This prospectively randomized study aimed to analyze the hemodynamic energy, vascular reactivity, endothelin-1 plasmatic levels and activity of erythrocyte nitric oxide synthase in patients undergoing cardiac surgery with either a pulsatile or non-pulsatile CPB.

## 2. Materials and Methods

### 2.1. Study Design and Patients Selection

Fifty-two patients aged ≥75 years undergoing aortic valve replacement at the Department of Cardiac Surgery of the University of Verona were prospectively enrolled in the study. Patients with indications for concomitant 1-vessel coronary artery bypass graft (CABG) or those with concomitant non-critical coronary artery disease (CAD) were also included. The exclusion criteria were as follows: indications for other concomitant heart procedures, myocardial infarction within the previous 1 month, insulin-dependent diabetes, renal dysfunction with preoperative creatinine level >2 mg/dL, history of systemic inflammatory disease, ongoing corticosteroid therapy, previous radiotherapy or chemotherapy, emergency procedures, patients in inotropic support, intra-aortic balloon pump (IABP) or circulatory assistance and re-operations.

All patients signed an informed consent, and the study protocol was approved by the University Ethical Committee (UEC). All patients were randomized by the Research Randomizer Program (http://www.randomizer.org; last accessed date: 22/02/2010) and divided into two perfusion-type groups. Twenty-seven patients underwent surgery with the blood pump set in a pulsatile mode (PP group), while twenty-five patients underwent surgery with the pump set in a non-pulsatile mode (NP group). The study was designed as a prospective randomized, parallel-group clinical study. Main demographic and clinical characteristics of the patients are reported in Table 1.

### 2.2. Anesthetic and Surgical Management

Anesthesia was provided according to a standardized protocol. Premedication consisted of oral diazepam 5–10 mg 2 h preoperatively. General anesthesia was induced with propofol infusion of 1–2 mg/kg and sufentanil of 0.03–0.05 µg/kg. Tracheal intubation was achieved with vecuronium 0.1mg/kg and the lungs were ventilated with air and oxygen (FiO2 = 0.5). The central venous catheter and a Swan–Ganz catheter (Arrow International,2400 Bernville Road, Reading, PA 19605, USA) were inserted in the right internal jugular or right subclavian vein and an indwelling bladder catheter for urine collection was routinely utilized. After anesthesia induction, the patients received 2.0 g of cefuroxim (or 600 mg of clindamycine when allergic to penicillin). Anesthesia was maintained with a propofol infusion of 2–4 mg/kg/hour and boluses of 0.3–0.5 µg/kg sufentanil and 0.05mg/kg vecuronium every 40 min. As far as fluid management, 3 mL/kg/hour of balanced saline solution was used to maintain the patient’s liquid requirements. Transfusion of packed red blood cells (RBC) was indicated at hemoglobin levels <7 g/L during CPB and at hemoglobin levels <9 g/L afterwards An activated clotting time greater than 480 s was produced by administration of 4 mg/kg heparin before starting CPB.

After aortic cross-clamping and transverse aortotomy, the aortic valve leaflets were excised, and a careful decalcification of the aortic annulus was carried out whenever necessary. Based on the attending surgeon’s choice, the following prostheses were implanted: Carpentier–Edwards Perimount Magna pericardial prosthesis (Edwards Lifesciences, Irvine, CA, USA) valve in 37 (93%) patients; Sorin Freedom Solo stentless valve (Sorin Biomedica, Saluggia, Italy) in 3 (7%) patients. Twelve patients underwent concomitant 1-vessel CABG procedure (6 patients in PP group and 6 patients in NP group). During CABG procedure, distal and proximal anastomosis (either arterial or venous) were performed with total CPB, aortic cross-clamp and cardioplegic heart arrest.

### 2.3. Perfusion Protocol

In both groups, the CPB circuit was primed with 1500 mL of balanced saline solution, while the flow was maintained at 2.4 L/min/m^2^. Myocardial protection during aortic cross-clamping was obtained through anterograde and retrograde Buckberg cardioplegic solution. The CPB was performed in normothermic conditions with mean arterial pressure (MAP) maintained between 50 and 80 mmHg. Hypotension (when lower than 50 mmHg) was corrected with norepinephrine bolus. After removal of aortic cross-clamp, the patient was warmed until nasopharyngeal temperature exceeded 36.5 °C and rectal temperature reached 35 °C. The infusion of 4 mg/kg of protamine was used to neutralize heparin at the end of CPB.

### 2.4. Circuit Components and Pump Settings

A new MEDOS Delta Stream DP3 centrifugal blood pump was used during CPB and was set in pulsatile (PP group) or non-pulsatile (NP group) mode. All the components of CPB circuit were equal between groups and were carefully selected according to their capacity to provide the maximum pulse delivery to the patient. The circuit consisted of a Maquet Quadrox-i Adult hollow-fibre “Softline™”-coated membrane oxygenator with an integrated heat exchanger, a Maquet Jostra Quart external screen-type arterial filter (pore size 40 µm), a corresponding “Softline™” coated tubing system with constant arterial line length from oxygenator to the aortic cannula of 145 + 10 cm and an Edwards 20.3-centimeter aortic cannula with a vented 3/8 in connection site (Luer Lock), 22–24 fr. In pulsatile mode, the pump was set at maximum rotation speed variation of 3500–8500 rpm with a pulse frequency of 60 bpm; start and stop points of the pulse were set at 20 and 80% of the pulse cycle, respectively.

### 2.5. Hydrodynamic Pump and Circuit Evaluation

The Shepard model was applied to evaluate the capacity of the pump to produce and to deliver pulsatile flow to the patient [10]. This model includes the hemodynamic energy gradient as a key point rather than the pressure gradient and it is actually described in terms of energy equivalent pressure (EEP) and surplus hemodynamic energy (SHE).

EEP was calculated according to the following formula: EEP =$\int_{t1}^{t2}fpdt/\int_{t1}^{t2}fdt$ (mm Hg), where *f* is blood flow, *p* is blood pressure and the product of flow and pressure represents hemodynamic power. The area under the power curve is represented by *∫fpdt* and the area under the flow curve by *∫fdt*. The EEP is a ratio of their magnitudes at the end of the pulse cycle. The measure unit of EEP is mmHg for easy comparison with the mean pressure. During PP-perfusion, the EEP is always higher than MAP and the difference between the 2 values characterizes pulsatility. This difference gives an idea of the extra energy transmitted by pulsatile flow to the patient and is expressed as SHE. SHE converts this energy difference into energy measure units by means of the experimentally obtained coefficient, 1332, and is calculated according to the following formula: SHE = 1332 (EEP − MAP) (ergs/cm^3^).

For a more precise EEP calculation, flow and pressure curves were recorded by external flowmeters (Transonic HT-110) and pressure transducers, respectively. Waves were recorded simultaneously during CPB at pump outlet, at post-oxygenator level, at the entrance of the aortic cannula and in the patients’ radial artery as shown in the previously published diagram [11]. As there was no technical possibility to put the flowmeter probe on the radial artery, the EEP of the patient was estimated indirectly utilizing the data from aortic cannula flowmeter probe and radial pressure transducer, taking into account the time difference (delay) between the flow wave recorded in the cannula and corresponding radial pressure wave. The delay used for calculations was set at 100 ms. The curves were recorded during 20-s intervals and digitally stored in the computer-based system for further analysis at the following 6 time-points during CPB: (1) after CPB onset, (2) 1 min, (3) 15 min, (4) 30 min of aortic cross-clamp, (5) rewarming, (6) after cross-clamp removal.

### 2.6. Hemodynamic Patients’ Evaluation

**Figure 1:**
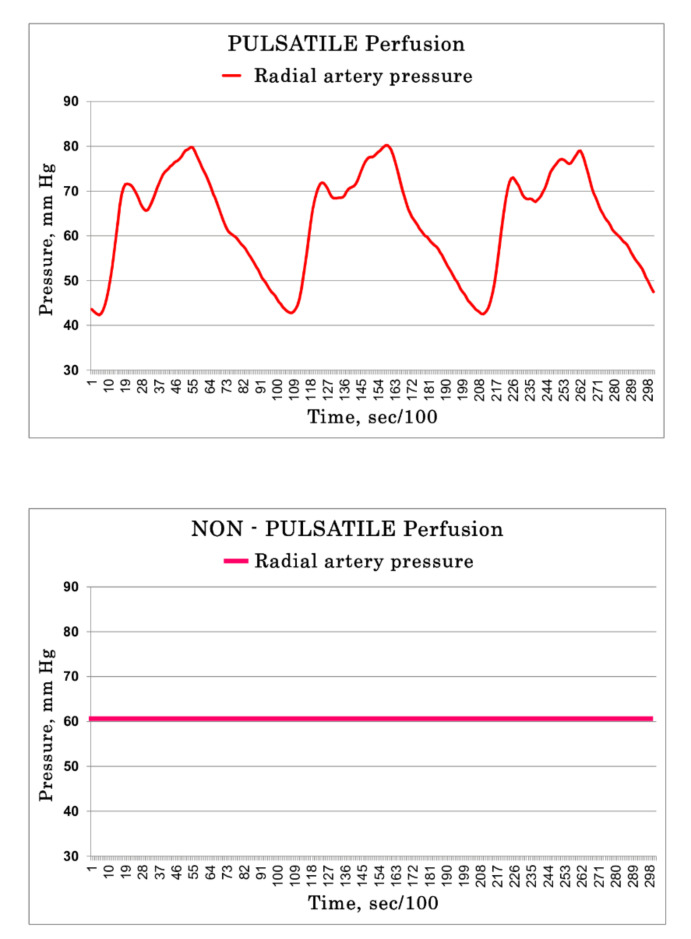
Examples of curve pressure over time in PP Group and in NP group.

Preoperative and postoperative patient hemodynamic evaluations were performed using the data from Swan–Ganz catheter including cardiac index (CI), mean pulmonary artery pressure (PAPm), wedge pressure, systemic vascular resistance (SVR) and pulmonary vascular resistance (PVR). During CPB, the flowmeter and radial artery pressure line curves were used (Figure 1). SVR and PVR were evaluated pre-operatively (15s after anesthesia induction) and the following 4 times after CPB: (1) before protamine administration; (2) 15s after protamine administration, (3) 2 h and (4) 18 h into Intensive Care Unit (ICU) stay. Patients’ SVR was also evaluated during CPB at the same 6 time-points with the pump hydrodynamic performance and was calculated according to the formula, SVR = 80(MAP – CVP)/CO, where MAP is mean arterial pressure; CVP is central venous pressure and is considered to be 0 mm Hg; and CO is cardiac output, which considered to be equal to the mean pump flow. Both SVR and PVR were further indexed by body surface area (BSA).

### 2.7. Hematologic and Metabolic Data Collection

A complete cells blood count was performed once before surgery, on arrival in ICU, at 2 h and 18 h into their ICU stay. Blood gas analysis (BGA), including pO2, pCO2, Hb, lactates and glycaemia, were performed on arrival in operating room, every 15 min during CPB, every 30 min afterwards in the operating room and every hour in the ICU. The lactates were monitored together with glycaemia in order to distinguish possible tissue hypoxia from primarily metabolic disturbances. CPB hemolysis rate was evaluated by means of free hemoglobin detection in plasma sampled just after CPB weaning. All intra- and postoperative transfusion requirements were recorded.

### 2.8. Endothelial Integrity Markers and Erythrocyte NOS Activity

Endothelial integrity was evaluated by means of plasmatic levels of von Willebrand Factor (vWF), intercellular adhesion molecule–1 (ICAM-1) and endothelin–1 (ET-1). Blood samples were taken at the following 4 times during perioperative period: (1) 10s after anesthesia induction; (2) just after sternal closure (3) 2 h and (4) 18 h after ICU arrival. The samples were anticoagulated with ethylenediaminetetraacetic acid (EDTA), centrifuged at 3600 rpm × 8 min, stored at −80 °C, and plasmatic markers were further determined using a high-sensitivity enzyme immunoassay (Dacopatts, Glostrup, Denmark). The nitric oxide activity was estimated indirectly by means of erythrocyte nitric oxide synthase (eNOS) activity analysis according to methods previously described [12]. The enzyme activity was estimated by measuring the conversion of L-[3H]-arginine to L-[3H]-citrulline and expressed as *pmol* of citrulline formed in 1 min by 1 mg of protein.

### 2.9. End-Points

Since progressive vasoconstriction and thus worse tissue perfusion is considered one of the most important biological effects of non-pulsatile CPB in our applied model, the primary endpoint was defined as any statistically differences between PP group and NP group in perioperative indexed systemic vascular resistance (SVRi).

Any significant differences between the groups in indexed pulmonary vascular resistance (PVRi), perioperative lactate levels, plasmatic levels of endothelin-1 and activity of erythrocyte endothelial-like nitric oxide synthetase (e-NOS) were defined as secondary endpoints.

### 2.10. Statistical Analysis

Sample-size estimation was performed by using the freely available G*Power 3.1.3 software package (Heinrich-Heine-Universitat Dusseldorf; Düsseldorf, Germany). Knowing, from one of previous studies [13], the mean difference in SVRi between the groups and assuming for our study the better outcomes in pulsatile group with mid-size effect, we estimate that, if α-error is taken of 0.05 and β-error of 0.10 (statistical power of 0.90) for a primary endpoint with foreseeable number of measures within each group of at least 4, a minimal number of 20 patients per group was required.

Data were checked for a normal distribution using the Kolmogorov–Smirnov test. Normally distributed continuous variables were described as mean ± SD and compared using the two-tailed Student’s t-test for paired groups or using an ANOVA test for multiple groups or repeated measures. Not normally distributed data were presented as median (25–75th percentiles) and compared using the Mann–Whitney *U*-test for paired groups, Wilcoxon test for paired repeated measures or Freedman test for multiple repeated measures. The frequencies of categorical variables were compared between groups using the chi-squared test. A simple linear regression model was used to analyze the relationship between eNOS and SHE in PP group. The correlation coefficient between variables was computed using the Pearson correlation coefficient. Statistical significance was accepted at *p* < 0.05. Statistical analysis was performed by using a commercially available SPSS version 17.0 (SPSS, Inc., an IBM Company, Chicago, IL, USA) software package.

## 3. Results

### 3.1. Hydrodynamic Pump and Circuit Evaluation

During both types of perfusions, the mean measured flow produced by the pump corresponded to the calculated one (4.4 ± 0.5 L/min of measured flow vs. 4.2 ± 0.5 L/min of calculated in the PP group; 4.3 ± 0.5 L/min of measured flow vs. 4.2 ± 0.5 L/min of calculated in the NP group) and the mean rotation velocity of the pump was similar. The MAP values within the circuit were comparable between the two groups and dropped progressively when measured at the pump outlet (219 ± 17 mm Hg in the PP group and 213 ± 38 mm Hg in the NP group, *p* = 0.57), at the post-oxygenator site (190 ± 22 and 172 ± 30 mmHg, respectively, *p* = 0.04) and at the entrance of the aortic cannula (107 ± 18 and 101 ± 17 mmHg, respectively, *p* = 0.31). The MAP in the radial artery was 55 ± 9 mm Hg in the PP group and 60 ± 13 mm Hg in the NP group, *p* = 0.15. The pulse pressure in the PP patients varied from 25 ± 6 to 32 ± 19 mmHg. In the NP group, there was no phasic flow or pressure variation, and the pulse pressure was always «0». During PP, the pressure oscillation in the circuit depended on the flow rate and decreased from the pump outlet to the aortic cannula. A flow rate of 3.5 L/min led to the maximum pressure variations at pump outlet from 22 to 360 mmHg and a flow rate of 5.5 L/—from 53 to 520 mmHg, respectively. The higher limits of blood pressure declared from the producers of the pump (600 mmHg) were never reached.

**Figure 2:**
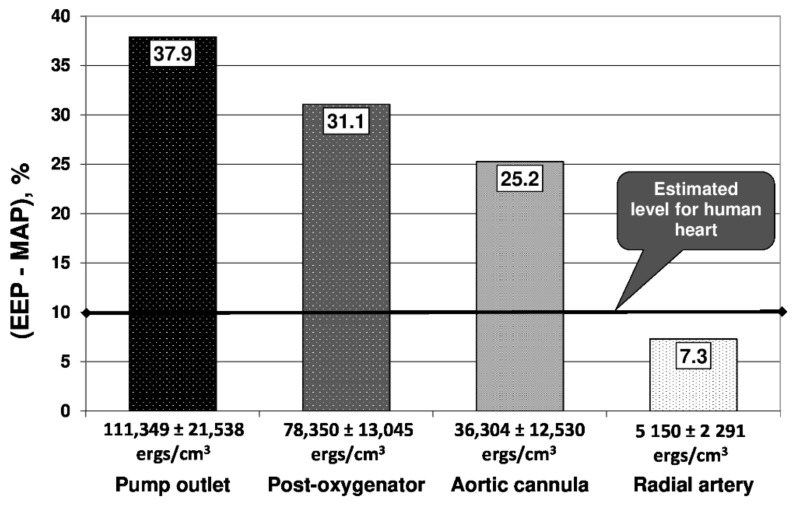
Hydrodynamic evaluation of “Blood pump—CPB circuit—Patient” system by means of EEP and SHE. Each diagram represents a mean percentage difference between EEP and MAP with a corresponding level of SHE (ergs/cm^3^) as a subtitle.

During NP, the EEP was always equal to the MAP at all the circuit levels, thus no SHE was ever produced. During PP, the EEP was 302 ± 12 mmHg at the pump outlet (37.9% higher than the MAP at the same level), 249 ± 25 mmHg at the post-oxygenator site (31.1% higher than the MAP), 134 ± 25 mmHg at the entrance of the aortic cannula (25.2% higher than the MAP) and 59 ± 10 mmHg in the patients’ radial artery (only 7.3% higher than the MAP). Even though the pump was able to produce a high-quality pulsatile flow with 111349 ± 21538 ergs/cm^3^ of SHE at its outlet, the circuit components damped down the “pulsatility” and significantly reduced the amount of surplus energy. The aortic cannula tip was a crucial point for the pulsatility drop with only 5150 ± 2291 ergs/cm^3^ of SHE evaluated for the patients’ radial artery (Figure 2).

### 3.2. Hemodynamic Analysis

**Figure 3:**
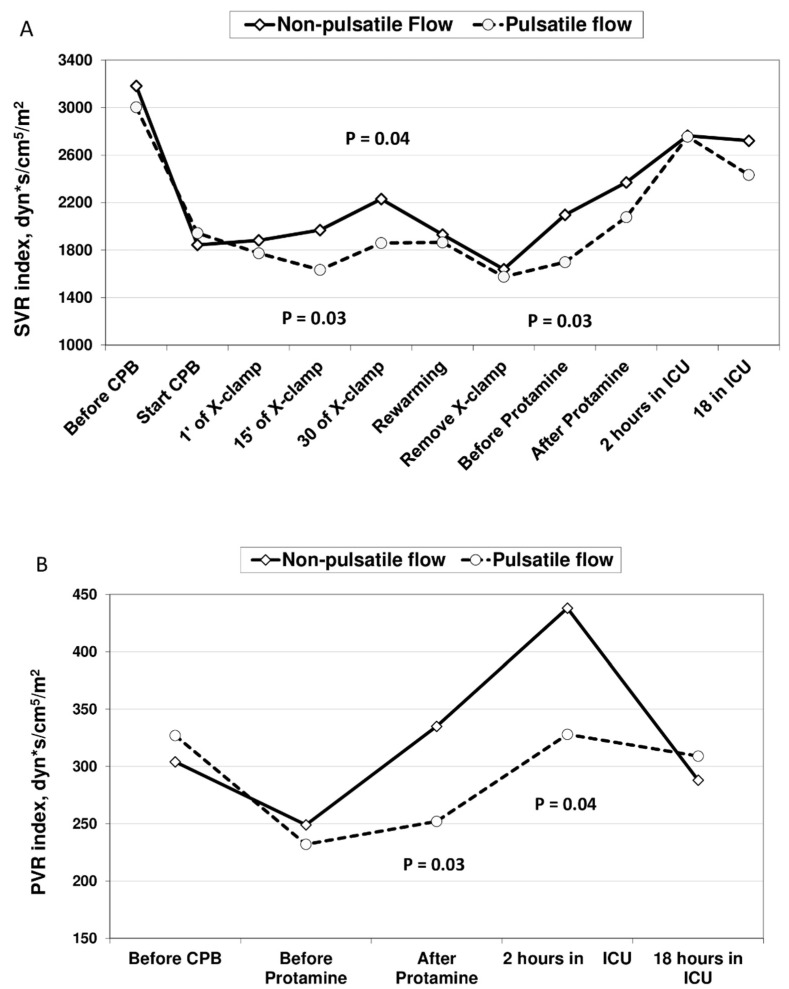
Perioperative profiles of indexed systemic (SVRi) and pulmonary (PVRi) vascular resistance. Panel (**A**) SVRi curve. Panel (**B**) PVRi curve.

The complete hemodynamic data obtained at six time-points during CPB are presented in Table 2, while the pre- and post-operative hemodynamic data are reported in Table 3. During the whole perfusion period, the flow rates remained constant without significant differences between the groups. The MAP was always maintained within the limits due to the perfusion protocol (range from 50 to 80 mm Hg) and was significantly different between the groups at 15 and 30 min of aortic cross-clamp. This pressure difference corresponded to the significant difference in SVRi calculated for the same time-points (Figure 3A). The perioperative hemodynamic analysis showed the significant postoperative improvement of the Cardiac Index (CI) in both groups without significant differences. No difference was found between the groups in terms of the post-perfusion MAP, PAPm and wedge values. SVRi was significantly lower in the PP group during the period between cross-clamp removal and protamine administration with further loss of difference after the protamine infusion (Figure 3A). PVRi was significantly lower in the PP group when it was measured after protamine administration and after 2 h in the ICU (Figure 3B).

During the CPB period, 18 (90%) patients from the PP group and 12 (60%) from the NP group (*p* = 0.03) required norepinephrine to correct arterial hypotension and maintain the target perfusion pressure. The median of the total intraoperative dosage of norepinephrine shots was 185 (60–320) µg in the PP group and 55 (0–158) µg in the NP group (*p* = 0.02). Postoperatively, nine (45%) of the PP patients and two (10%) of the NP patients (*p* = 0.01) required vasopressor/inotropic support consisting of adrenalin, noradrenalin or both for a period between 6 to 48 h.

### 3.3. Endothelial Integrity Markers and Erythrocyte NOS Activity

**Figure 4:**
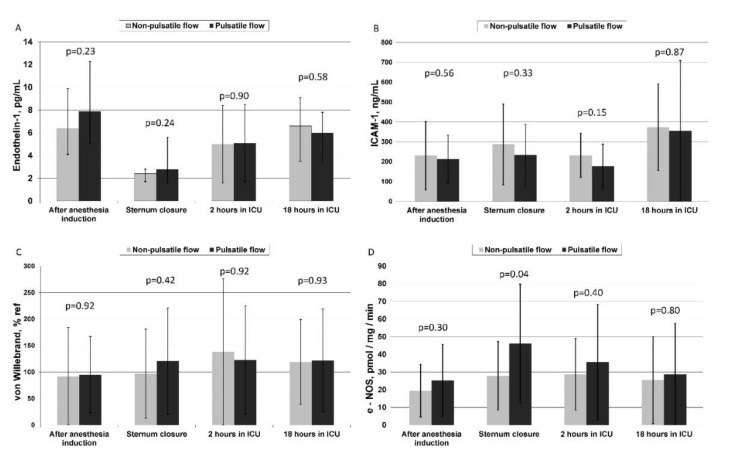
Diagrams of perioperative plasma levels of endothelial integrity markers and erythrocyte NOS activity during pulsatile and non-pulsatile perfusion Panel (**A**). Plasma levels of Endothelin-1 (ANOVA group-time interaction, *p* = 0.28) Panel (**B**). Plasma levels of ICAM-1 (ANOVA group-time interaction, *p* = 0.94). Panel (**C**). Plasma levels of von Willebrand Factor (ANOVA group-time interaction *p* = 0.77). Panel (**D**). Erythrocyte e-NOS activity (ANOVA group–time interaction *p* = 0.08).

ET-1 showed maximal plasma levels before surgery in both groups. It dropped down significantly after the CPB and increased gradually for up to 18 h in the ICU. No significant difference was found between the groups at any sampling time-point (Figure 4A). ICAM-1 and vWF had a slight tendency to augment their plasma levels at 2 h (for vWF) and 18 h (for ICAM-1) postoperatively without any difference between the groups (Figure 4B,C).

**Figure 5:**
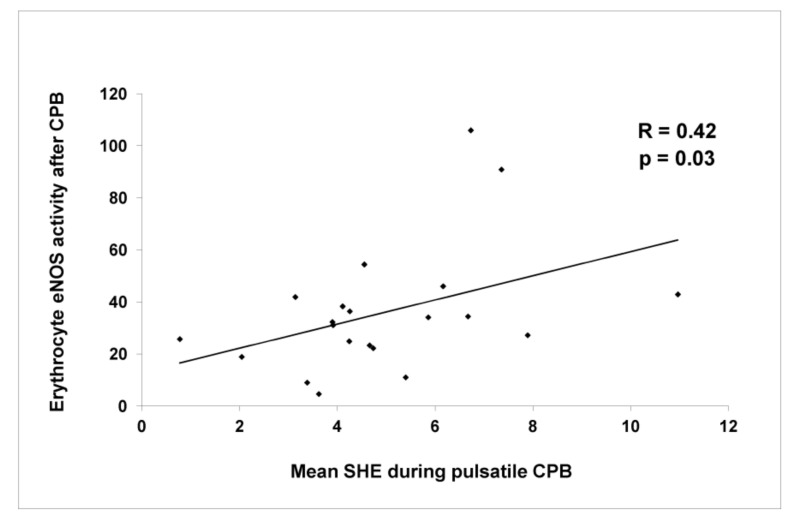
Diagrams of erythrocyte NOS activity during pulsatile and surplus hemodynamic energy (SHE).

The eNOS showed a significant activity increment at the end of the CPB with its further lowering after 18 postoperative hours. At the end of the CPB, a significant difference in enzyme activity was found between the PP and NP groups (46.2 ± 33.6 vs. 27.9 ± 19.4 pmol/mg/min, respectively, *p* = 0.04) (Figure 4D). Furthermore, in the PP group, eNOS activity after the CPB was found to have a significant positive correlation with SHE measured during the CPB (R = 0.42; *p* = 0.03) (Figure 5).

### 3.4. Metabolic Markers of Perfusion Quality

The mean CPB time (84 ± 22 min in the PP group and 86 ± 20 min in the NP group, *p* = 0.76) and the aortic cross-clamp time (64 ± 19 and 67 ± 18 min, respectively, *p* = 0.54) were similar between the two groups. The adequate quality of perfusion in both the PP and NP groups was confirmed by the similar plasma lactate levels at any timepoints during the CPB, as well as at 18 h postoperatively. No significant differences were found in the plasma creatinine levels, intraoperative and early postoperative urinary output and furosemide requirements. The difference in the postoperative glomerular filtration rate calculated as creatinine clearance did not reach statistical significance between the groups (61 ± 35 mL/min in PP group vs. 47 ± 16 mL/min in NP group, *p* = 0.12). On the other hand, when comparing the pre- and post-operative values of creatinine clearance, no significant difference was found in the PP group (71 ± 26 vs. 61 ± 35 mL/min *p* = 0.95), while a statistically significant decrement was observed in the NP group (70 ± 28 vs. 47 ± 16 mL/min *p* = 0.001) (Table 4).

The two groups were statistically similar in terms of the preoperative hemoglobin (Hb) level (11.3 ± 1.9 g/dL in the PP group and 11.7 ± 1.9 g/dL in the NP group, *p* = 0.47), intraoperative hemodilution (minimum Hb level during CPB was 8.1 ± 1.3 and 8.3 ± 1.3 g/dL, respectively, *p* = 0.51) and intraoperative RBC transfusion requirements (248 ± 283 and 221 ± 228 mL, respectively, *p* = 0.89). Higher values of plasma free hemoglobin were observed in the PP group (probably as a consequence of the higher hemolysis rate due to the Venturi effect of negative pressure that occurs in the limiting diameter of the aortic cannula, which accounts for a very high flow) (37 ± 18 and 28 ± 12 mg/dL in the PP group and the NP group, respectively, *p* = 0.10); however, they did not meet the requirements of statistical significance. Furthermore, this observed values did not lead to any significant differences in the postoperative RBC transfusion rates (378 ± 328 and 338 ± 268 mL, respectively, *p* = 0.75) and fresh frozen plasma transfusions (450 ± 488 and 616 ± 587 mL, respectively, *p* = 0.33), as well as in postoperative drainage loss (578 ± 334 and 536 ± 416 mL, respectively, *p* = 0.72).

### 3.5. Clinical Results

The hospital mortality was 5% (two patients). From the PP group, an 83-year-old patient died on the 57th postoperative day because of sepsis; the other patient from the NP group died on the 25th postoperative day because of pneumonia and respiratory failure. All the other patients were discharged successfully and showed a satisfactory clinical outcome at follow-up.

No difference was found in the two groups regarding intubation time (14 ± 5 h for the PP group and 15 ± 6 h for the NP group, *p* = 0.71), ICU stay (26 (20–66) and 23 (20–47) hours, respectively, *p* = 0.68), hospital stay (9 (8–12) and 9 (8–14) days, respectively, *p* = 0.82) or postoperative complications rates. The early complications that required a prolonged (>24 h) ICU stay included postoperative bleeding (four patients from the PP group with no reoperations and four patients from the NP group with two re-sternotomies performed, *p* = 0.15), low cardiac output syndrome (two patients from the PP group), gastro-intestinal bleeding (one patient from the NP group), transient ischemic attack (one patient from the NP group) and paroxysmal atrial fibrillation (one patient from each group).

## 4. Discussion

To the best of our knowledge, this is the first prospective randomized clinical study that compared the actual hemodynamic energy and vascular reactivity of pulsatile versus non-pulsatile perfusion during a CPB. The main finding of this study was that patients perfused with a pulsatile flow had an augmented release of vasodilative molecules, reduced systemic and pulmonary vascular resistances with better organs perfusion, which resulted in a preserved post-CPB renal glomerular filtration rate. However, patients with PP-perfusion during the CPB required higher perioperative vasopressor support.

The study aimed to find evidence for or to reject the superiority of short-term PP over NP in patients undergoing cardiac surgery. Besides the lack of clinical trials, the major reasons for debating this issue include the high heterogeneity of criteria and models for pulsatile flow description, the variety of technological solutions and the different biological criteria for perfusion efficacy [4]. As the amount of hemodynamic energy delivered to the patients’ circulation was shown to correlate with the intensity of the biological effects of perfusion [4,13], the Shepard’s surplus energy model was applied to describe pulsatile perfusion [10]. The centrifugal pumps are considered to be superior to traditionally used roller pumps, since they provide better biocompatibility and hemodynamic performance in pulsatile mode [4]. Selected circuit components, especially designed, were also shown to provide the maximum of pulse conductance [4,14,15]. During NP, the EEP and MAP were always equal and no surplus energy hemodynamic was produced. During PP, at the pump outlet, the EEP was at an average of 38% higher than the MAP; while passing along the circuit, the blood flow progressively lost its “pulsatility”. This phenomenon was related to the loss of energy damped by the oxygenator, arterial line and aortic cannula and finally, in the patients’ radial artery, the difference between the EEP and MAP was only 7.3%, approximately three-quarters of the 10–12% physiological difference estimated for the human heart [3,4]. Thus, all the biological and clinical effects observed during PP were achieved with those three-quarters of physiological “pulsatility”. The existing technological limits of effective pulse delivery during CPB were stressed by recent studies with IABP applied to create a pulsatile flow. In contrast to the controversial results of “classical” PP studies, the majority of IABP studies showed the explicit benefits of PP in terms of inflammatory response, coagulation effects, metabolic changes and clinical outcomes [16]. The IABP catheter placed in the aorta produces a significantly higher amount of hemodynamic energy without damping the influence of the CPB circuit and aortic cannula.

In contrast to the results of Sezai et al. [17], in the studied groups no significant differences were found in terms of perioperative lactates and endothelial integrity markers. Lactates had a slight tendency to augmentation during CPB and early afterward, but remained within the protocolled limits.

Several studies described the tendency to increase hemolysis rates with the augmentation of pump speed variance during the pulse cycle, mainly with roller pumps [18,19]. In our experience, the maximal pump speed variation was set in order to obtain the maximum amount of hemodynamic energy. This led to the appearance of moderate levels of free plasmatic hemoglobin. A slightly higher amount of free hemoglobin in the PP group did not reach statistical significance comparing with the NP group. However, it did not result in higher postoperative transfusion rates or higher postoperative anemia.

Although the most important theoretical mechanism of positive PP effects is considered to deal with microcirculation, vascular resistance and critical capillary closing pressure, only a few papers dedicated to perioperative systemic vascular resistance monitoring are available. There are few animal models with controversial results [20,21] and four major clinical papers were found [13,22,23,24]. Taylor et al. measured patient’s peripheral vascular resistance before and after CPB and found significantly lower post-CPB values in the PP group along with better preserved plasma angiotensin II levels [13]. This was followed by a study by Philbin et al., who found that PP significantly attenuates the vasopressin and catecholamine stress response to CPB [22]. In contrast, Frater et al. monitored SVR during and just after CPB together with plasma cortisol, ADH and renin activity and failed to find any differences [23]. Louagie et al. studied the biggest group of 100 patients and found no difference between the groups in terms of perioperative systemic vascular resistance, lactate levels, renal functions and postoperative mortality [24].

Along with the existing technological limitations for energy transfer, there were few strong confounding factors, masking or counterbalancing the endothelial effects of PP. The endothelium reacts not only to the mechanical stimuli and energy transfer but also to the biochemical stimuli derived from various types of perioperative therapy, transfused blood products, vasopressor agents, etc. The pulse itself, even having perfect hemodynamic characteristics, does not eliminate systemic inflammation (SIRS) as a consequence of blood contact with the circuit foreign surfaces. Thus, all kinds of endothelium proteins involved in the inflammatory reactions, including ICAM and vWF, may be influenced dominantly by SIRS and much less by the hemodynamic properties of the flow.

CPB itself was shown to augment plasmatic concentrations of ET-1 in the early postoperative period [18]. The same study found a significantly lower ET-1 increment during the PP flow compared to the NP flow. In our study, both groups demonstrated very high initial plasma levels of ET-1 that decreased significantly just after CPB. It could be explained by the aortic stenosis that required a high level of preoperatively vasoconstriction to maintain adequate perfusion pressure. Once the stenosis was removed, the factors responsible for vasoconstriction went down, even though it was influenced by CPB. The absence of the difference between the two groups could be explained by the significantly higher usage of vasopressors during PP, as ET-1 is known to be very sensitive to these drugs.

The PP group showed significantly lower SVRi during all of the aortic cross-clamp period until the rewarming phase. The observed hemodynamic effects were likely caused by plasma nitric oxide (NO) molecules synthesized by both endothelial and erythtocyte NOS. NO is known as the most potent and sensible vasodilator but is hardly detectable due to its ultra-short half-life [12]. On the other hand, the human RBC are known to express an active and functional endothelial-type NOS and its activity is supposed to be comparable with “traditional” endothelial-cells NOS [25]. Moreover, it was shown recently that CPB activates erythrocyte NOS [26] and higher shear-stress applied to the erythrocyte wall can stimulate additional NO production by RBC [27]. In our study, the PP group demonstrated significantly higher erythrocyte NOS activity at the end of CPB compared to the NP group. Furthermore, the amount of eNOS activity was resultingly correlated to the SHE produced during PP flow. We could assume, therefore, that increasing the shear-stress from the pulsatile blood flow provided similar effects on NO production when either applied to the RBC wall or to the vascular endothelium.

The probable explanation for the marked reduction in SVRi in the NP group during the rewarming period and after the removal of the aortic cross-clamp is the competitive impact of rewarming as a physical phenomenon that counteracts the “vasoconstrictor” effects of a non-pulsatile flow and provides a vasodilator effect. In contrast, after protamine administration, a significant increment of SVRi was observed in the PP group. It is explained by the influence of higher vasopressor usage in the patients of the PP group during the early post-CPB period.

When PVRi was considered, it was higher after protamine administration in the NP group than in the PP group, as well as after two hours into their ICU stay. It reached statistically significant values in terms of the time–group interaction for multiple repeated measures (ANOVA). The lungs are known to be the most ischemic organ during CPB, thus could be extremely sensitive to the quality of perfusion through the bronchial arteries. If there had been some positive effects of PP during perfusion time, it could result in a lower amount of toxic (pro-inflammatory, post-ischemic) products in pulmonary circulation after the aortic cross-clamp removal. This, probably, led to a better preserved pulmonary vascular tone and lower post-protamine vasoconstriction. In contrast, studies by Engels et al. did not demonstrate a beneficial effect of PP on postoperative lung function and lung injury biomarkers [28].

Finally, the glomerular filtration rate in the early postoperative period was better preserved in the PP group, as we have already demonstrated in a previous study. However, PP flow did not result in other major clinical advantages [29]. We hypothesize that in spite of the presence of current technological limitations for “pulse” delivery, the obtained SHE was enough to provide some perceptible effects on the patients’ circulation during short-term CPB. Unfortunately, some of the effects could be excessive in terms of vasodilation, thus leading to a higher dosage of vasopressors. On the other hand, the PP benefits are counterbalanced with the other unfavorable CPB effects that are actually not modifiable.

Considering its beneficial impact on the endothelial and microvascular system [7,8], pulsatile flow should be further investigated in extracorporeal perfusion. As an example, it could be of interest especially if long-term circulatory support is considered. Pulsatile long-term mechanical supports are being used less often due to their large size, the thrombogenic surfaces and their poor long-term durability. On the other hand, even if malfunction complications have been drastically reduced with the current continuous-flow machines, the long-term complications of the non-pulsatile flow on the microvascular system are well known and described (i.e., shearing of von Willebrand factor multimers, loss of mucosal integrity from oxidative stress, development of arteriovenous malformations, increased angiogenesis) [30,31,32]. Therefore, further studies on this topic should be welcomed.

The study limitations consisted of a small number of patients and the presence of some confounding factors that are hardly modifiable when the endothelial effects of perfusion are studied. Preoperative therapy and intraoperative blood transfusions could act as such a confounding factors. This did not allow for the comparison of postoperative complication rates or adverse outcomes. Few extrapolations on major clinical results could be taken due to the lack of sufficient statistical power. However, the present study was specifically targeted on the analysis of the hemodynamic effects of either type of perfusion. Another limitation is related to the extremely selective population that included only AVR patients. Therefore, further studies should be conducted in order to analyze the pulsatile effect in other cohorts of patients.

## 5. Conclusions

To date, a pulsatile flow with similar physiological characteristics is hardly reproducible with the existing technologies of blood delivery during CPB. However, it results in significantly lower systemic and pulmonary vascular resistance and plasma lactate levels during the early post-perfusion period. Furthermore, the activity of endothelium-dependent vasodilators and vasoconstrictors during pulsatile perfusion depends on the amount of hemodynamic energy transmitted to the patient. Further investigations are required to investigate the clinical impact of the higher vasopressor dosage during pulsatile perfusion.

## Figures and Tables

**Table 1:** Patients’ main demographic and clinical data.

	PP	NP	*p*-Value
Number of patients	27	25	–
Age (years)	80 ± 3.1	80 ± 3.4	0.56
Gender (M/F)	11/16	14/11	0.27
EUROScore (units)	5.9 ± 1.3	5.6 ± 1.5	0.45
BSA (m^2^)	1.8 ± 0.2	1.8 ± 0.2	0.97
Arterial hypertension (pts) (%)	18 (67)	19 (76)	0.46
Diabetes mellitus (pts) (%)	7 (26)	5 (22)	0.27
Coronary artery disease (pts) (%)	14 (52)	10 (40)	0.39
COPD (pts) (%)	3 (11)	4 (17)	0.52
Permanent AF (pts) (%)	7 (26)	8 (35)	0.50
Renal insufficiency ^1^ (pts) (%)	2 (7)	3 (12)	0.58
Preop ACE-inhibitors (pts) (%)	13 (48)	13 (52)	0.78
Preop statins (pts) (%)	18 (67)	16 (64)	0.84

**Legend:** PP: pulsatile perfusion; NP: non-pulsatile perfusion; M: male; F: female; BSA: body surface area; pts: patients; COPD: chronic obstructive pulmonary disease; AF: atrial fibrillation preop: preoperative; ACE: angiotensin converting enzyme. ^1^ Renal insufficiency: serum creatinine between 1.2–2 mg/dL.

**Table 2:** Hemodynamic profile during cardiopulmonary bypass.

		Start CPB	1s of Cross-Clamp	15s of Cross-Clamp	30s of Cross-Clamp	Re-Warming	Cross-Clamp Removal	Means for all CPB Period	ANOVA *p* (Group/Time Interaction)
**MAP (mmHg)**	PP	57 ± 15	58 ± 14	51 ± 11	58 ± 12	58 ± 14	51 ± 11	56 ± 9.0	0.03
	NP	59 ± 12	61 ± 15	64 ± 19 **	69 ± 19 *	61 ± 18	57 ± 12	62 ± 12 *	
**Flow (L/m)**	PP	4.3 ± 0.7	4.4 ± 0.6	4.3 ± 0.7	4.3 ± 0.6	4.3 ± 0.7	4.2 ± 0.8	4.3 ± 0.6	0.64
	NP	4.4 ± 0.6	4.4 ± 0.5	4.4 ± 0.5	4.3 ± 0.4	4.4 ± 0.5	4.3 ± 0.5	4.4 ± 0.4	
**EEP (mmHg)**	PP	61 ± 17	62 ± 16	54 ± 12	63 ± 13	62 ± 16	54 ± 13	60 ± 10	0.10
	NP	59 ± 13	60 ± 16	63 ± 20	68 ± 21	60 ± 19	53 ± 10	61 ± 13	
**EEP-MAP (%)**	PP	6.4 ± 2.7	6.6 ± 3.3	7.4 ± 2.9	7.7 ± 2.7	6.8 ± 3.4	5.5 ± 2.6	6.8 ± 2.4	<0.01
	NP	0 ± 0.0 **	0 ± 0.0 **	0 ± 0.0 **	0 ± 0.0 **	0 ± 0.0 **	0 ± 0.0 **	0 ± 0.0 **	
**SHE (ergs/cm^3^)**	PP	5131 ± 3070	5462 ± 3563	4951 ± 2050	6121 ± 2668	5638 ± 3551	3891 ± 2194	5259 ± 2231	<0.01
	NP	0 ± 0.0 **	0 ± 0.0 **	0 ± 0.0 **	0 ± 0.0 **	0 ± 0.0 **	0 ± 0.0 **	0 ± 0.0 **	
SVRi (dyn*sec/cm^3^/m^2^)	PP	1913 ± 576	1844 ± 528	1677 ± 405	1897 ± 412	1930 ± 572	1757 ± 645	---	0.04
	NP	1910 ± 454	1948 ± 523	2057 ± 640 *	2264 ± 692 *	1972 ± 644	1849 ± 444	---	
**Pulse pressure (mm Hg)**	PP	32 ± 19	28 ± 11	25 ± 6	31 ± 9	31 ± 16	28 ± 17	31 ± 8.2	---
	NP	0 ± 0.0	0 ± 0.0	0 ± 0.0	0 ± 0.0	0 ± 0.0	0 ± 0.0	0 ± 0.0	

**Legend:** CPB: cardiopulmonary bypass; PP: pulsatile perfusion; NP: non-pulsatile perfusion; MAP: mean arterial pressure; EEP: energy equivalent pressure; SHE: surplus hemodynamic energy; SVRi: systemic vascular resistance indexed; * *p* < 0.05 when PP and NP groups values were compared; ** *p* < 0.001 when PP and NP group values were compared.

**Table 3:** Hemodynamic profile before and after cardiopulmonary bypass.

		Before Surgery	Before the Protamine	15s after the Protamine	2 h in ICU	18 h in ICU	ANOVA *p* (Group/Time Interaction)
**MAP (mm Hg)**	PP	75 ± 12	67 ± 10	70 ± 9.6	79 ± 9.5	82 ± 12	0.98
	NP	80 ± 16	70 ± 9.1	73 ± 10	83 ± 14	84 ± 11	
**Cardiac index (L/min/m^2^)**	PP	1.8 ± 0.5	2.7 ± 0.5	2.5 ± 0.5	2.2 ± 0.4	2.5 ± 0.6	0.02
	NP	1.9 ± 0.5	2.4 ± 0.7	2.2 ± 0.6*	2.2 ± 0.5	2.4 ± 0.5	
**PAPm (mm Hg)**	PP	20 ± 5.2	21 ± 4.2	21 ± 5.3	23 ± 4.1	23 ± 6.2	0.28
	NP	20 ± 3.9	21 ± 4.6	22 ± 5.0	25 ± 5.5	21 ± 4.5	
**Wedge pressure (mm Hg)**	PP	13 ± 3.9	14 ± 2.7	13 ± 4.2	15 ± 3.4	14 ± 4.3	0.22
	NP	12 ± 3.4	14 ± 3.7	13 ± 4.3	14 ± 4.1	12 ± 3.4	
SVRi (dyn*sec/cm^3^/m^2^)	PP	3161 ± 919	1781 ± 468	2024 ± 607	2571 ± 810	2326 ± 454	0.39
	NP	3271 ± 1122	2237 ± 752 *	2477 ± 755 *	2713 ± 745	2566 ± 618	
PVRi (dyn*sec/cm^3^/m^2^)	PP	326 ± 119	226 ± 96	248 ± 116	302 ± 152	284 ± 126	0.01
	NP	317 ± 127	257 ± 93	329 ± 98 **	418 ± 141 **	302 ± 102	

**Legend:** PP: pulsatile perfusion; NP: non-pulsatile perfusion; MAP: mean arterial pressure; PAPm: mean pulmonary artery pressure; SVRi: systemic vascular resistance indexed; PVRi: pulmonary vascular resistance indexed. * *p* < 0.05 when PP and NP group values were compared. ** *p* < 0.001 when PP and NP group values were compared.

**Table 4:** CPB-time, cross-clamp time and perioperative profiles of lactates, glycaemia, creatinine clearance.

		PP	NP	*p*-Value
CPB time (min)		84 ± 22	86 ± 20	0.76
Cross-clamp time (min)		64 ± 19	67 ± 18	0.54
Lactates (mmol/mL)	Before CPB	0.8 ± 0.3	0.8 ± 0.2	0.96
	At 30s of cross-clamp	1.1 ± 0.4	1.2 ± 0.3	0.39
	At cross-clamp removal	1.3 ± 0.5	1.4 ± 0.5	0.85
	After CPB	1.4 ± 0.5	1.4 ± 0.4	0.77
	At ICU arrival	1.4 ± 0.4	1.7 ± 0.9	0.22
	2 h in ICU	1.3 ± 0.5	1.7 ± 1.1	0.15
	18 h in ICU	1.9 ± 0.9	2.0 ± 1.1	0.70
Glycaemia (mmol//mL)	Before CPB	6.0 ± 1.5	5.7 ± 1.9	0.63
	At 30s of cross-clamp	7.9 ± 2.3	7.8 ± 2.3	0.81
	Al cross-clamp removal	8.4 ± 2.8	7.8 ± 1.5	0.37
	After CPB	7.9 ± 1.9	7.7 ± 2.3	0.79
	At ICU arrival	6.9 [6.0–8.4]	6.2 [5.8–8.2]	0.43
	2 h in ICU	8.5 ± 2.6	7.5 ± 1.5	0.09
	18 h in ICU	9.1 ± 1.4	9.4 ± 1.7	0.44
Pre-op plasma creatinine (µmol/L)		91 ± 21	99 ± 31	0.32
Pre-op CCr, 18 h (mL/min/1.73 m^2^)		71 ± 26	70 ± 28 *	0.71
Post-op CCr, 18 h (mL/min/1.73 m^2^)		61 ± 35	47 ± 16 *	0.06

**Legend:** PP: pulsatile perfusion; NP: non-pulsatile perfusion; CPB: cardio-pulmonary bypass; ICU: intensive care unit. Pre-op: preoperative; CCr, 18h: creatinine clearance calculated in 18 h and normalized for 1.73m^2^ of body surface; Intra-op: intraoperative; * *p* < 0.05 when pre- and postoperative values were compared.

## Data Availability

All data are contained in the manuscript.
